# Distal Femoral Replacement and Extensor Mechanism Repair Reinforced With Synthetic Mesh for Distal Femur Fracture With Patellar Ligament Avulsion

**DOI:** 10.1016/j.artd.2022.04.001

**Published:** 2022-05-23

**Authors:** Charles Powell, Kristopher Sanders, Neal Huang, Luis Felipe Colón, Colton Norton

**Affiliations:** a Department of Orthopaedic Surgery, Erlanger Health System Center, Chattanooga, TN, USA; b Department of Orthopaedic Surgery, University of Tennessee at Chattanooga, Chattanooga, TN, USA

**Keywords:** Extensor mechanism reconstruction, Synthetic mesh, Distal femoral replacement, Supracondylar femur fracture, Case report

## Abstract

Acute patellar ligament disruption in the setting of a distal femur fracture is an uncommon presentation with a variety of treatment options available to the practitioner. The following case report presents an 85-year-old female with a highly comminuted supracondylar distal femur fracture with intercondylar extension and a soft-tissue avulsion of the patellar ligament insertion discovered intraoperatively. A detailed technique review for acute patellar ligament repair with suture anchors and synthetic mesh reinforcement in the setting of distal femoral replacement is then provided. One-year follow-up revealed an intact extensor mechanism with minimal extensor lag and a painless gait. Surgeons faced with such a unique, complex problem may consider mesh augmentation of an acute patellar ligament repair while performing distal femoral replacement.

## Introduction

Distal femur fractures can be devastating injuries in the elderly, resulting in morbidities and mortality similar to those with geriatric femoral neck fractures [1,2]. Highly comminuted fractures involving the articular surface and metaphyseal region impose a significant task upon the orthopedic surgeon. If open reduction with internal fixation (ORIF) is not viable, an alternative option is prosthetic replacement using a distal femoral replacement (DFR). This technique was first described in 1982 and is now widely accepted and utilized among orthopedic surgeons [3]. Patellar ligament disruption in the setting of a closed supracondylar femur fracture is exceptionally rare [4]. The following work describes a combination of 2 well-described surgical techniques for acute repair of a patellar ligament soft-tissue avulsion off the tibial tubercle using suture anchors bolstered with synthetic mesh in the setting of a DFR for a distal femur fracture. One-year follow-up was completed with excellent clinical outcomes and patient satisfaction. The purpose of this case report is to present a complex problem and detailed surgical technique for future surgeons to employ and successfully treat such patients.

## Case history

**Figure 1:**
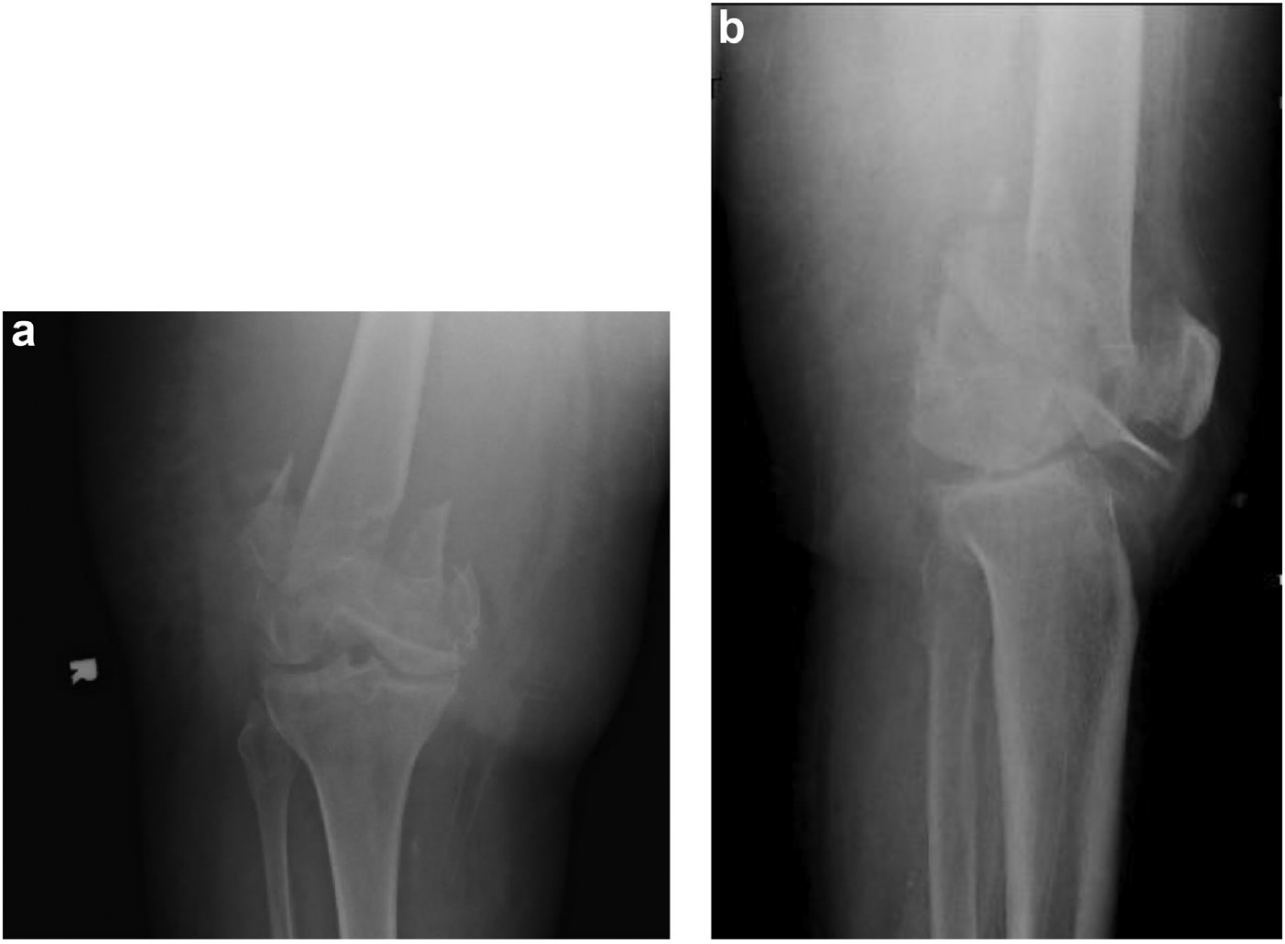
Anteroposterior (a) and lateral (b) injury radiographs demonstrating supracondylar intercondylar distal femur fracture with intercondylar extension with significant comminution of the articular and metaphyseal segments. Insall-Salvati ratio of 1.5.

Consent was obtained from the patient for the writing of this manuscript and publication. The patient was an 85-year-old female community ambulator without assisted devices who was involved in a head-on motor vehicle collision. Pertinent medical history included insulin-dependent diabetes mellitus. On initial physical exam, the patient complained of right knee and foot pain with significant swelling and obvious deformity without open wounds. She was found to be neurovascularly intact distally. Plain radiographs of the right knee revealed a highly comminuted and shortened supracondylar distal femur fracture with intercondylar extension as well as a Chopart fracture dislocation of the ipsilateral foot (Fig. 1). Proximal tibial skeletal traction was placed, and the Chopart dislocation reduced and splinted. The case was reviewed by several orthopedic traumatologists that determined her to be a poor candidate for ORIF due to significant articular and metaphyseal comminution. A referral was placed to an orthopedic joint reconstruction surgeon for consideration of a DFR.

### Surgical technique

**Figure 2:**
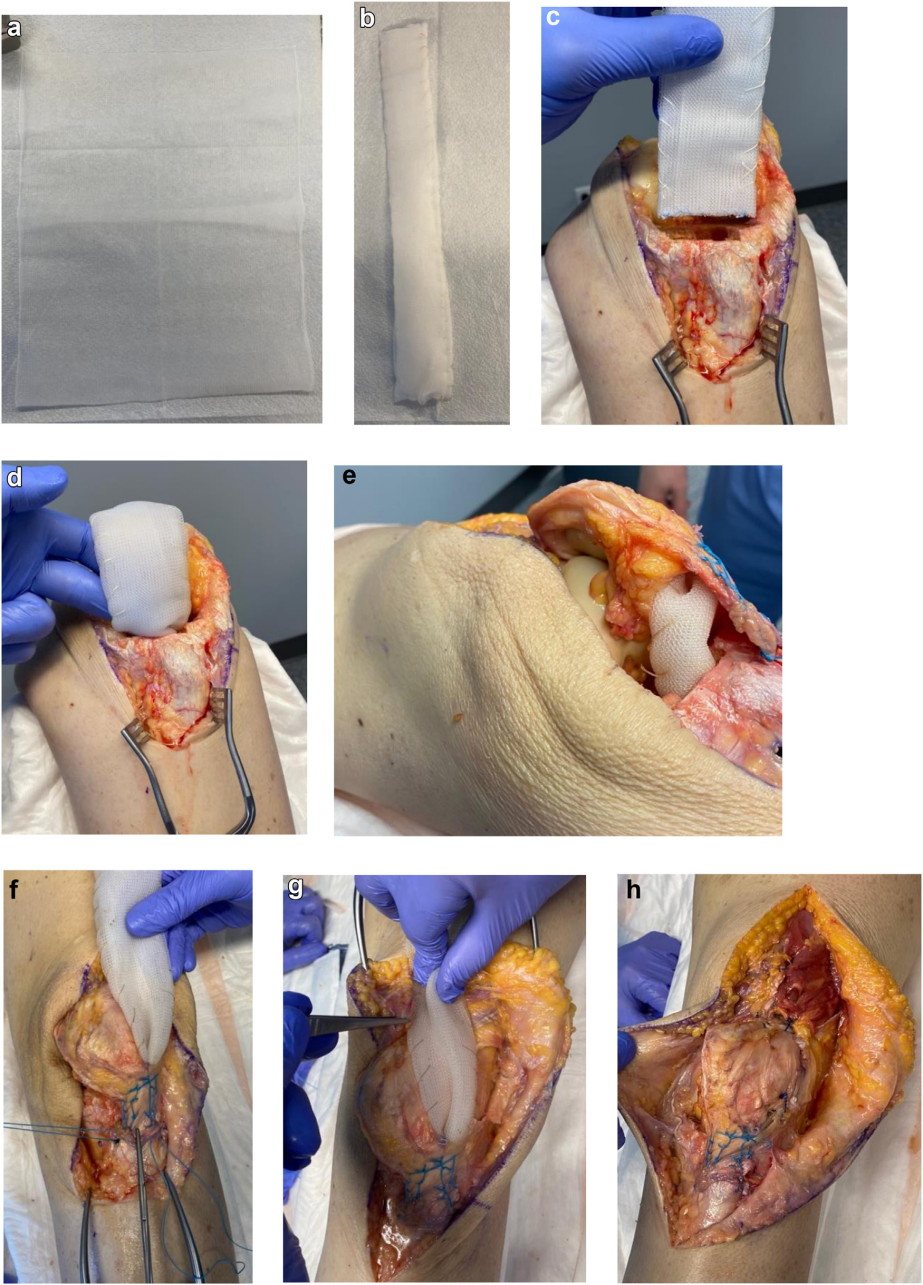
Surgical technique: (a) Bard Mesh, 10 in × 14 in (25 cm × 35.5 cm). (b) The mesh was folded 10 layers thick and approximately 2 cm wide and held with nonabsorbable suture. (c) An anterior cortical notch was formed with a rongeur approximately the width of the mesh and approximately 5 mm in length. (d) Two to five centimeter of mesh was cemented into the canal with the final tibial component. (e) The mesh was then tunneled between the retropatellar fat pad and ligament exiting through the lateral retinaculum. (f) Two suture anchors were placed medial and lateral to the tibial tubercle and Krakow stitched to the patellar ligament and tensioned, returning the ligament to its native footprint. (g) The mesh was then passed proximally through a lateral retinacular tunnel advancing proximally, superficial to the quadriceps tendon securing the mesh along its path with single-stranded polyethylene suture. (h) The retinacular tunnel is closed. Additionally, the vastus medialis oblique is mobilized, brought laterally covering the mesh overlying the quadriceps tendon, and secured to the vastus lateralis with single-stranded polyethylene suture.

Once adequately stabilized and resuscitated, the patient was taken to the operating room for DFR. Intraoperative images were not obtained. However, a cadaveric specimen was utilized to recreate the patellar ligament avulsion and simulate suture anchor repair with synthetic mesh augmentation as detailed in Figure 2. An anterior midline incision was utilized. Upon further dissection, her patellar ligament soft tissue was found to be completely stripped from the tibial tubercle in its entirety, which was not previously recognized on injury films. A medial parapatellar arthrotomy to include a prior traumatic retinacular and capsular tear was completed. Greater than 7 independent articular fragments were identified and subperiosteally excised. Further dissection revealed similar comminution of the metaphyseal segment with fragments removed in a similar fashion.

The distal femur metadiaphyseal region was exposed allowing a freshening cut to be carried out proximal to the metaphyseal comminution followed by reaming and trialing of the femoral component. Once satisfied, attention was turned to the tibial cut and preparation followed by trialing of the hinged components. Soft-tissue tensioning, gaps, leg length, and alignment were found to be appropriate. The patella was then measured and prepared. Attention was then turned to fixation of the synthetic mesh, which was a Bard Mesh, 10 in × 14 in (25 cm × 35.5 cm) by Becton, Dickinson and Company (BD) medical technology company, Franklin Lakes, NJ (Fig. 2a) [5]. The original description of the employed technique was described by Browne and Hanssen, specifically in the intramedullary position [6]. The mesh was folded 10 layers thick and approximately 2 cm wide and held with nonabsorbable suture (Fig. 2b). The proximal tibia was prepared for mesh placement within the intramedullary canal using a proximal metaphyseal reamer. Additionally, an anterior cortical notch matching the approximate width of the mesh and less than 5 mm in length was formed with a rongeur allowing anterior egress of the mesh from the eventual intramedullary position (Fig. 2c). Confirmation of adequate preparation was obtained by placing the mesh into the canal with the trial tibial component. Both intramedullary canals and bony surfaces were prepared for cementation followed by placement of cement restrictors. The mesh and tibial implant were then cemented into position with 2 to 5 cm of graft seated into the canal (Fig. 2d). This was held in place until hard with excess cement removed. Once cement hardening was confirmed, traction was applied to the mesh confirming fixation within the canal, allowing the surgeon to elevate the tibia independently from the surgical table. The femoral and patellar components were cemented into position using a second package of cement, the polyethylene liner impacted into position, and the hinge mechanism assembled. The tourniquet was deflated, and hemostasis was obtained.

The mesh was then tunneled between the infrapatellar fat pad and ligament exiting through the lateral retinaculum to create soft-tissue interposition between the mesh and the tibial components and polyethylene liner (Fig. 2e). Two suture anchors were then placed on the medial and lateral borders of the tibial tubercle. Krakow sutures were placed through the patellar ligament while providing inline distal traction to the ligament (Fig. 2f). Once secured, anatomic approximation of the patellar ligament to its footprint was achieved. The knee was ranged to 60 degrees of flexion to ensure the extensor mechanism functioned as a single unit as well as to assess for individualized potential for advancement of range-of-motion restrictions postoperatively similar to that described by Browne and Hanssen [6].

The mesh was then passed proximally through a lateral retinacular tunnel advancing proximally, superficial to the quadriceps tendon (Fig. 2g). The mesh was secured along its path with single-stranded polyethylene suture. The retinacular tunnel was closed over the mesh. Finally, the vastus medialis oblique is brought laterally to cover the proximal mesh and secured to vastus lateralis (Fig. 2h). The proximal arthrotomy was closed in a standard fashion. Again, the extensor mechanism was assessed to 60 degrees of flexion with no excess tension applied to the patellar ligament insertion allowing ascertainment of previously mentioned parameters as well as to ensure appropriate patellar tracking after mesh placement. Intrawound antibiotics and hemostatic agents were injected into the joint as well as placed superficial to the arthrotomy followed by a layered closure and finally application of incisional negative pressure wound therapy. Closed reduction and splinting in anatomic alignment of the Chopart joint were carried out.

**Figure 3:**
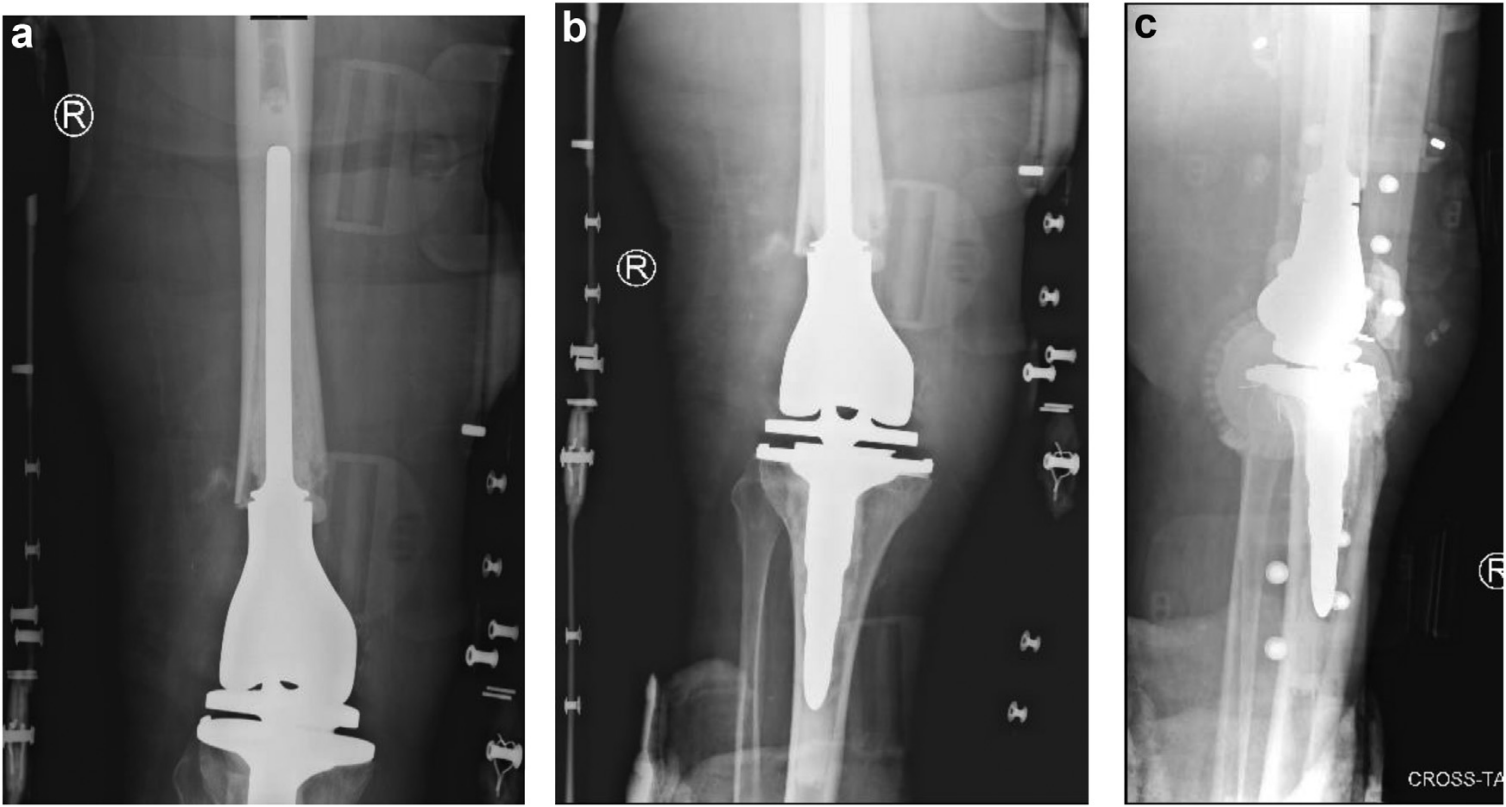
Anteroposterior (a and b) and lateral (c) 2-week postoperative radiographs demonstrating cemented distal femoral replacement with maintenance of alignment, rotation, and fixation. Insall-Salvati ratio of 1.18.

**Figure 4:**
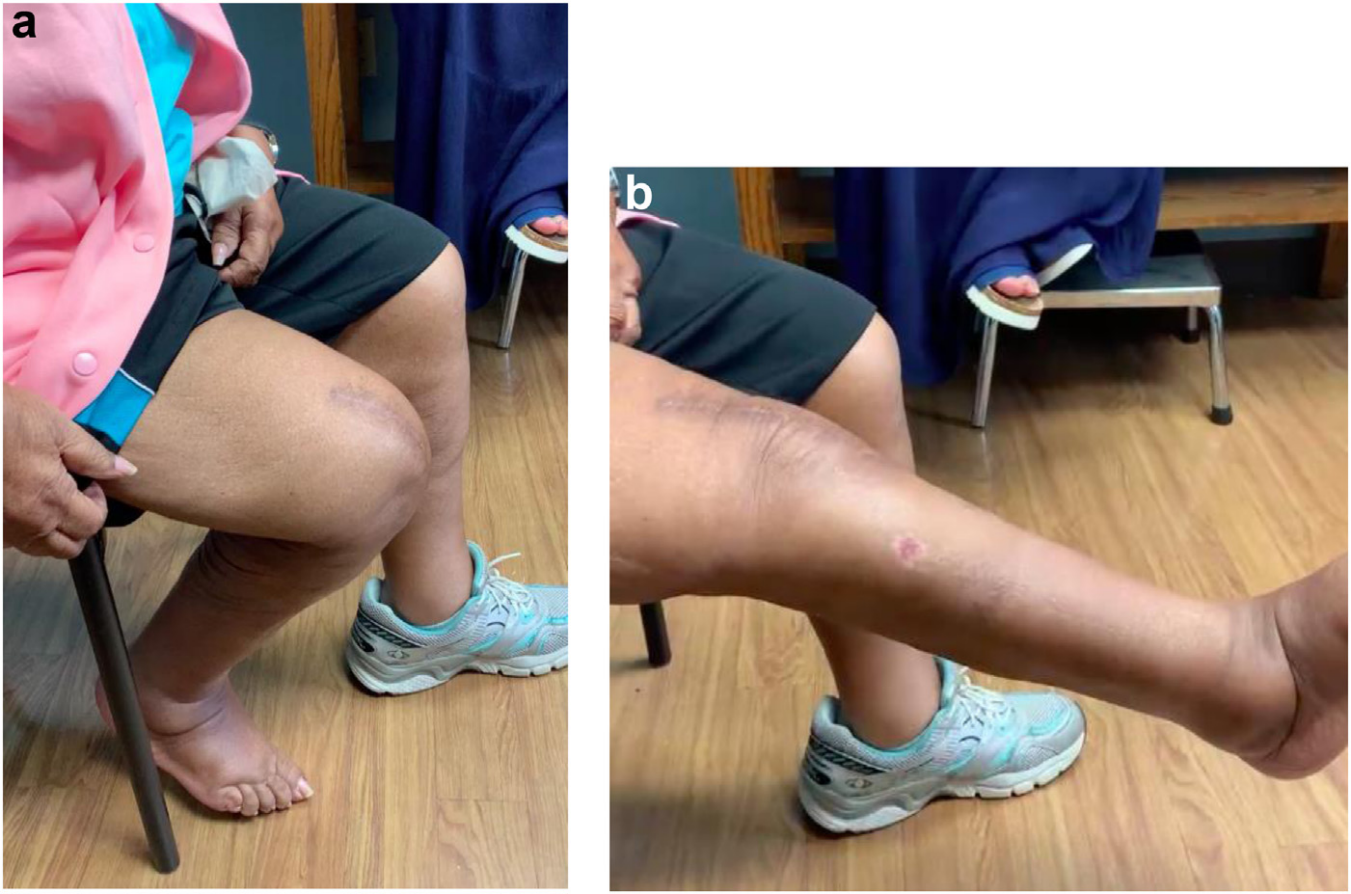
Photographs demonstrating (a) active flexion to approximately 130 degrees and (b) active extension to approximately 5 degrees from full extension at one-year follow-up appointment.

**Figure 5:**
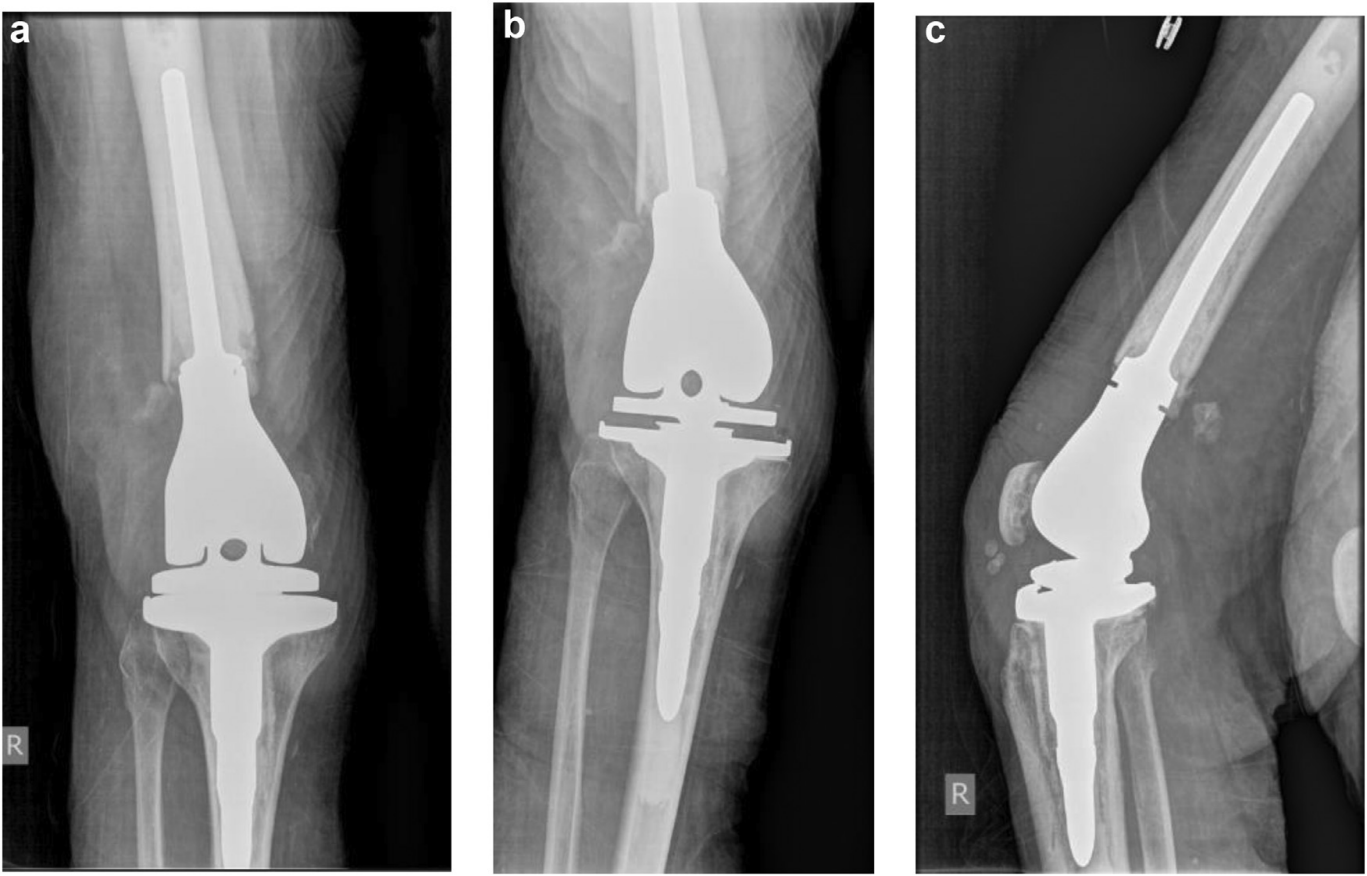
Anteroposterior (a and b) and lateral (c) 1-year postoperative radiographs demonstrating cemented distal femoral replacement with maintenance of alignment, rotation, and fixation. Insall-Salvati ratio of 1.22.

The patient was made non-weight-bearing due to the Chopart dislocation. Neither a hinge knee brace locked in full extension with no range of motion nor quadriceps isometric exercises restrictions were applied. While many surgeons advocate casting postoperatively, the primary investigator elected to utilize a hinge knee brace for the following reasons: excellent patient reliability demonstrated preoperatively, easier access for direct wound care purposes, avoidance of potential soft-tissue complications from long-term casting, and lighter weight allowing for increased ease with postoperative mobilization. Deep vein thrombosis and antibiotic surgical prophylaxis were initiated. She was neurovascularly intact postoperatively, following postoperative restrictions, and discharged home once she met appropriate criteria. The patient was seen at approximately 2 weeks postoperatively with pristine incisions. Radiographs revealed components to be well aligned, sized, and fixed with an Insall-Salvati ratio of 1.18 (Fig. 3). Foot radiographs revealed maintenance of reduction. The hinge knee brace was continued, locked in full extension with application of a short leg cast and continued non-weight-bearing restrictions. At 6 weeks postoperatively, radiographs were unchanged. The patient could maintain active knee extension with no extensor lag. Non-weight-bearing restrictions were continued. The patient was allowed active knee flexion to 30 degrees, advancing 10 degrees per week, with no active knee extension or passive flexion. The hinge knee brace was locked in extension at night. At 3 months, the patient’s passive knee range of motion was 0 to 45 degrees, and there was no extensor lag with active extension. Radiographic assessment remained unchanged. The patient was made weight-bearing with full active and passive range of motion as tolerated. At 4 months, the patient was ambulating with a rolling walker in a boot with passive knee range of motion of 0 to 110 degrees with less than 5 degrees of extensor lag. The patient was transitioned to an athletic shoe with a scheduled 1-year postoperative follow-up. At 1 year, the patient was ambulating with the use of a rolling walker while out of the house. Physical exam revealed active flexion and extension from 130 degrees to −5 degrees from full extension (Fig. 4). Radiographs revealed maintained alignment and fixation of the femoral and tibial components as well as integrity of the extensor mechanism (Fig. 5). Annual follow-up was scheduled.

## Discussion

DFR is a viable option for distal femur fracture, with studies comparing surgical fixation to arthroplasty finding similar outcomes and overall complication rates [[7], [8], [9], [10], [11]]. Specific types of complications, however, vary between the 2 procedures. Potential complications of DFR include extensor mechanism disruption despite being intact at the index procedure, later failure with loosening, prosthetic joint infection, and limited salvage options if treatment fails or major complications develop. Complications of ORIF, in contrast, include nonunion, malunion, knee stiffness, and compromised function, with the latter 2 likely related to prolonged non-weight-bearing following the operation [12]. A benefit of DFR is the allowance of immediate postoperative full weight-bearing and decreased time to mobilization. Due to the presented patient’s ipsilateral Chopart dislocation, weight-bearing restrictions were required for 3 months postoperatively. It is unknown how this would have affected the patient’s outcome.

Other considerations with our patient presentation include the technique for repair of her extensor mechanism disruption. In chronic cases, primary repair has largely been abandoned and replaced by reconstructive methods such as medial gastrocnemius flap reconstruction, alloplastic cord, allograft, and synthetic mesh [[6], [13], [14], [15]]. In contrast, acute presentation allows for direct primary repair. In the setting of significant soft-tissue stripping and retinacular and capsular destruction as that found with distal femoral fracture, the surgeon may wish to bolster the acute repair using described reconstructive techniques with synthetic mesh. Additionally, due to the known complication of extensor mechanism disruption following arthroplasty, it may be prudent to include a secondary fixation construct following direct repair. The literature describing proximal tibial modular endoprosthetic reconstructions comparing direct reattachment of the extensor mechanism to mesh augmented reattachment showed a lower incidence and smaller degree of extensor lag with mesh reconstruction [16]. Additionally, when compared with allograft, the use of synthetic mesh has shown equivalent success of reconstruction and knee outcome scores while potentially eliminating pitfalls of allograft including availability, graft mismatch to host, immune reaction, disease transmission, and cost [17].

Primary repair of a patellar ligament avulsion from its tibial tubercle insertion is possible in the acute setting. When significant soft-tissue stripping is present or a large arthrotomy is performed for arthroplasty, bolstering of the primary repair can be completed with synthetic mesh. For those completing DFR for distal femoral fractures, having synthetic mesh readily available is wise as patellar ligament disruption may be missed during preoperative planning as occurred in the presented case. This repair allows range-of-motion limitations to be gradually decreased at 6 weeks postoperatively, with excellent clinical outcomes and patient satisfaction at 1 year.

## Summary

This case report describes a combination of 2 well-described techniques that could aid orthopedic surgeons in addressing such a unique scenario. Future studies on this topic should focus on prospectively comparing this technique with previously described methods for extensor mechanism reconstructions in patients undergoing arthroplasty for fracture.

## Conflict of interest

The authors declare that there are no conflicts of interest.

For full disclosure statements refer to https://doi.org/10.1016/j.artd.2022.04.001.

## Informed patient consent

The author(s) confirm that informed consent has been obtained from the involved patient(s) or if appropriate from the parent, guardian, power of attorney of the involved patient(s); and, they have given approval for this information to be published in this case report (series).

## Appendix

1. An 85-year-old female presents with a highly comminuted distal femur fracture following a high-speed motor vehicle crash. Clinical exam is significant for a palpable defect at the insertion point of the patellar ligament. The decision is made to proceed with acute distal femoral replacement. Treatment of the associated injury shown in the Figure A can best be accomplished by
1. Allograft
2. Medial gastrocnemius flap reconstruction
3. Alloplastic cord
4. Suture anchor repair with synthetic mesh
5. Nonoperative care with knee immobilizer postoperatively.

Discussion: The above radiograph reveals a highly comminuted distal femoral fracture with disruption of the extensor mechanism. Answers A, B, and C are typically completed for chronic patellar ligament reconstruction. Answer E is inappropriate for extensor mechanism disruption in the setting of DFR.

2. An 85-year-old female presents with a highly comminuted distal femur fracture following a high-speed motor vehicle crash (Figure A). Clinical exam is significant for a palpable defect at the insertion point of the patellar ligament. She is found to have a patellar ligament disruption at its tibial insertion intraoperatively. The decision is made to proceed with acute distal femoral replacement with direct repair with mesh augmentation. Postoperative restriction should include
1. Non-weight-bearing for 6 weeks
2. Aggressive range of motion immediately
3. Continuous passive motion machine once wounds have healed
4. Full extension until 6 weeks postoperatively with graduate return
5. Hinge knee brace set 0-30 degrees immediately

Discussion: The above radiograph reveals a highly comminuted distal femoral fracture with disruption of the extensor mechanism repaired with suture anchor and mesh augmentation. Answer D has been shown to protect the repair with knee range of motion of 0 to 110 degrees with less than 5 degrees of extensor lag at 1 year.

3. An 85-year-old female presents with a highly comminuted distal femur fracture following a high-speed motor vehicle crash (Figure A). Clinical exam is significant for a palpable defect at the insertion point of the patellar ligament. She is found to have a patellar ligament disruption at its tibial insertion intraoperatively. The decision is made to proceed with acute distal femoral replacement with direct repair using mesh augmentation. Fixation of the mesh distally best can be accomplished by
1. Bone tunnels distally in the tibia with suture fixation to the mesh
2. Two double loaded anchors
3. Suture fixation to periosteum and anterior compartment fascia
4. Cementing into the canal with the tibial component
5. Cerclage wire

Discussion: Answer D has been shown to provide strong distal fixation of synthetic mesh without impacting tibial component placing. One can ensure it fits into the canal with the component while trialing. Fixation can then be tested intraoperatively once the cement mantle is hardened by simply lifting the mesh and the tibia along with it.

## Appendix A Supplementary data

Conflict of Interest Statement for Norton

Conflict of Interest Statement for Powell

Conflict of Interest Statement for Sanders

Conflict of Interest Statement for Colon

Conflict of Interest Statement for Huang
