# Supplementary material for: Distal Femoral Replacement and Extensor Mechanism Repair Reinforced With Synthetic Mesh for Distal Femur Fracture With Patellar Ligament Avulsion
Source: Arthroplast Today. 2022 May 23;16:31–8. doi: 10.1016/j.artd.2022.04.001 (PMC9136045; doi:10.1016/j.artd.2022.04.001)
Supplement: Conflict of Interest Statement for Powell [file mmc2.docx]

# CONFLICT OF INTEREST STATEMENT

***American Association of Hip and Knee Surgeons***

(Adopted from the American Academy of Orthopaedic Surgeons disclosure statement)

The following form **must be filled out completely and submitted by each author (example, 6 authors, 6 forms).**

**All items require a response. If there is no relevant disclosure for a given item, enter "*None*.”**

Distal Femoral Replacement and Extensor Mechanism Repair Reinforced with Synthetic Mesh for Distal Femur Fracture with Patellar Ligament Avulsion: Case Report and Surgical Technique

1. Royalties from a company or supplier (The following conflicts were disclosed)

none

2. Speakers bureau/paid presentations for a company or supplier (The following conflicts were disclosed)

none

3A. Paid employee for a company or supplier (The following conflicts were disclosed)

none

3B. Paid consultant for a company or supplier (The following conflicts were disclosed)

none

3C. Unpaid consultants for a company or supplier (The following conflicts were disclosed)

none

4. Stock or stock options in a company or supplier (The following conflicts were disclosed)

none

5. Research support from a company or supplier as a Principal Investigator (The following conflicts were disclosed)

none

6. Other financial or material support from a company or supplier (The following conflicts were disclosed)

none

7. Royalties, financial or material support from publishers (The following conflicts were disclosed)

none

8. Medical/Orthopaedic publications editorial/governing board (The following conflicts were disclosed)

none

9. Board member/committee appointments for a society (The following conflicts were disclosed)

none

**Each author must sign AND print or type his/her name, date and submit a separate form**

In addition, one BLINDED Conflict of Interest form (no author names used) should be submitted per manuscript with all author disclosures.

Charles W Powell Charles W Powell 2/4/2022

Author Name (Print or Type) Author Signature Date
